# A Programmable *Escherichia coli* Consortium via Tunable Symbiosis

**DOI:** 10.1371/journal.pone.0034032

**Published:** 2012-03-30

**Authors:** Alissa Kerner, Jihyang Park, Audra Williams, Xiaoxia Nina Lin

**Affiliations:** 1 Department of Biomedical Engineering, University of Michigan, Ann Arbor, Michigan, United States of America; 2 Department of Chemical Engineering, University of Michigan, Ann Arbor, Michigan, United States of America; 3 Center for Computational Medicine and Bioinformatics, University of Michigan, Ann Arbor, Michigan, United States of America; University of Massachusetts, United States of America

## Abstract

Synthetic microbial consortia that can mimic natural systems have the potential to become a powerful biotechnology for various applications. One highly desirable feature of these consortia is that they can be precisely regulated. In this work we designed a programmable, symbiotic circuit that enables continuous tuning of the growth rate and composition of a synthetic consortium. We implemented our general design through the cross-feeding of tryptophan and tyrosine by two *E. coli* auxotrophs. By regulating the expression of genes related to the export or production of these amino acids, we were able to tune the metabolite exchanges and achieve a wide range of growth rates and strain ratios. In addition, by inverting the relationship of growth/ratio vs. inducer concentrations, we were able to “program” the co-culture for pre-specified attributes with the proper addition of inducing chemicals. This programmable proof-of-concept circuit or its variants can be applied to more complex systems where precise tuning of the consortium would facilitate the optimization of specific objectives, such as increasing the overall efficiency of microbial production of biofuels or pharmaceuticals.

## Introduction

Microbial consortia, or groups of interacting microbial populations, are found in diverse natural and synthetic environments where they can perform complex tasks such as assisting mammals in food digestion [1] or wastewater treatment [2]. For example the human gut microbiota, populations of microbes living in the human gastrointestinal tract, perform many important functions for their human hosts. These include harvesting important nutrients, synthesizing vitamins, detoxifying foreign substances, supporting the immune system, and participating in renewal of the gut epithelium [1]. Since nature often relies on interacting microbes rather than on one superbug, it follows that humans may succeed in achieving complex tasks by imitating her through the use of synthetic microbial consortia. The prime advantage of consortia is their ability to complete tasks that would be too difficult for one organism, such as the co-fermentation of glucose and xylose from lignocellulosic biomass [3]. Other advantages of synthetic consortia include: the ability to compartmentalize pathways so that they can be individually optimized, the ability to simplify the optimization process by the division of labor, and the possibility that the system can be tightly controlled by external signals [4].

Genetic circuits have been used in synthetic microbial consortia to construct programmable patterns [5], to render various relationships among consortium members [6], [7], [8], and to engineer artificial biofilms [9], [10]. These systems were created mainly to mimic and investigate the dynamics or other properties of natural consortia and are very well suited for this task. On the other hand, there have been few efforts on developing genetic circuits to enable precise tuning of microbial consortia. The capability of precise regulation could be a crucial property for synthetic microbial consortia in various applications. For example, when two hexose- and pentose-specialists are used for optimal utilization of hexose and pentose sugars derived from lignocellulosic biomass, different ratios of the two strains might be desired depending on the composition of the feedstock. In this work we have constructed a tunable, synthetic consortium of two *E. coli* auxotrophs which cross-feed and support each other when grown in co-culture. The ability to fine-tune this forced symbiosis is made possible via regulating the exchange of two amino acids (tryptophan and tyrosine) in a continuous manner. Depending on the amount of inducers added to the medium, we are able to obtain a wide range of growth rates and co-culture composition. This system is a proof-of-concept circuit that demonstrates the possibility of programming and tuning a synthetic microbial consortium.

## Results

### Basic scheme: growth rate and ratio can be tuned in an inter-dependent pair of microbes

**Figure 1:**
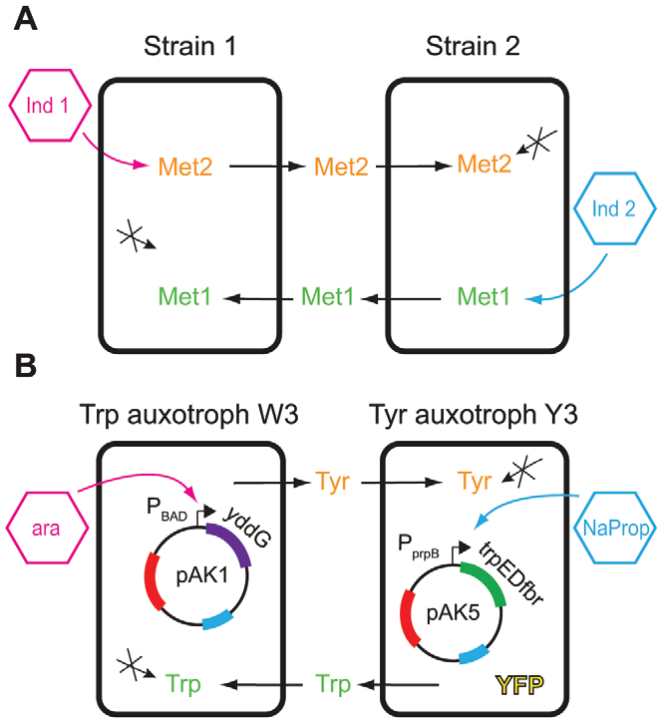
Basic schematic of the tunable cross-feeding circuit. (**A**) In this general design, inducer 1 and inducer 2 control the export of metabolites 1 and 2, respectively. The two auxotrophs must cross-feed in order to survive in the minimal medium. (**B**) In our specific implementation, two *E. coli* auxotrophic strains exchange tryptophan (Trp) and tyrosine (Tyr). The forced symbiosis is controlled by plasmids pAK1 (in the Trp auxotroph, W3) and pAK5 (in the Tyr auxotroph, Y3). Plasmid pAK1 contains gene *yddG* behind the tunable promoter P_BAD_, and pAK5 contains *trpEDfbr* behind P_prpB_ (Methods). Strain Y3 is tagged with yellow fluorescent protein (YFP).

We have designed a genetic circuit, based on metabolic cross feeding, to enable continuous tuning of the growth rate and composition of a synthetic two-member microbial consortium. A schematic of our tunable circuit is shown in Figure 1A, wherein two auxotrophs are forced to depend upon each other for growth. In a minimal medium lacking key metabolites, these two microbes do not grow unless they exchange the required nutrients in an efficient manner. Previous work suggested that such a pair of inter-dependent microbes, when grown together in a batch co-culture and given enough time, reaches a pseudo steady state characterized by unchanging concentrations of cross-fed metabolites and consortium composition (Reppas, Lin, *et al.*, manuscript in preparation). Serial passaging experiments with auxotroph pairs indicated that this was indeed the case (data not shown). At this pseudo steady state the two consortium members have the same growth rate. In addition, this growth rate of the co-culture and the ratio of the two microbes are determined solely by each auxotroph's export rate of its partner's required metabolite and the auxotroph's growth requirement for the metabolite it demands, as illustrated mathematically below (see Methods for details):
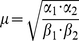
(1)

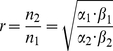
(2)where μ represents the co-culture growth rate, r is the ratio of the cell density of Auxotroph 2 (n_2_) versus that of Auxotroph 1 (n_1_). α_1_ denotes Auxotroph 1's export rate of Metabolite 2 required by Auxotroph 2 (e.g. with a unit of mmol/gDM*hr) and β_1_ is Auxotroph 1's cellular requirement for Metabolite 1 (e.g. with a unit of mmol/gDM). Similarly, α_2_ and β_2_ describe Auxotroph 2's corresponding properties. Based on this theoretical prediction, it should therefore be possible to control the co-culture growth rate and the ratio of the two microbes by manipulating either the auxotrophs' export of the two cross-fed metabolites (i.e. α_1_ and α_2_) or their cellular requirement for the metabolites (i.e. β_1_ and β_2_). The former strategy appeared more straightforward and we further decided to explore the usage of chemical inducers to regulate the synthesis and transport pathways related to the export of the two cross-fed metabolites (Figure 1A).

### Implementation with a pair of *E. coli* tryptophan and tyrosine auxotrophs

In this work, we implemented the basic scheme described above with a specific pair of tryptophan and tyrosine *E. coli* auxotrophs (Figure 1B). To tune the α values experimentally, genes related to Trp and Tyr export were cloned behind inducible promoters that can produce a continuous range of expression levels.

In order to tune the export rate (α) of either amino acid, one could manipulate the actual export of the molecule or attempt to overproduce the molecule, assuming that the extra Trp or Tyr would be exported and passed on to the partner strain. So far only one aromatic amino acid exporter has been identified, the inner membrane protein YddG which, when over-expressed in *E. coli*, has been shown to increase the export of all three aromatic amino acids from corresponding, engineered Phe-, Tyr-, or Trp-producers [11]. Since this protein increases the amount of Tyr in the medium more than Trp (3-fold increase versus 1.5-fold), it was chosen for regulating the export of Tyr in strain W3. The *tyrR* gene has also been knocked out in W3, since this may increase the carbon flux through the tyrosine pathway by up-regulating genes upstream of *tyrAB* [12] and possibly increase the chance that manipulating YddG production will have an effect (in our preliminary studies with YddG manipulation there was an insignificant effect until *tyrR* was knocked out).

On the other side of the circuit, we decided to over-produce Trp in strain Y3 to increase the export of Trp. The Trp biosynthetic pathway has been extremely well studied and several mechanisms to render pathway enzymes feedback-resistant have also been elucidated [13], [14], [15]. Based on this previous knowledge, a feedback-resistant mutant of anthranilate synthase, which is encoded by genes *trpED* and catalyzes the first step in the pathway of Trp biosynthesis, was chosen for over-expression and regulation. The gene construct *trpEDfbr* was commercially synthesized with the mutation S40F inserted into the *trpE* gene. The S40F mutation has been found to render *E. coli* resistant to Trp analogues such as 5-methyltryptophan and the affected residue was involved in a potential Trp-binding site [16], [17].

To induce and tune the above genes chosen for regulation of Trp/Tyr export, it would be best to choose two inducible promoters that function simultaneously and do not exhibit crosstalk. There are a number of inducible promoters available for use in *E. coli* but not all of them are tunable or able to produce a large expression gradient [18]. After comprehensive review of related literature, two compatible promoters were chosen: the arabinose-inducible promoter *P_BAD_* and a recently created propionate-inducible promoter, *P_prpB_*. These two promoters both produce a large gradient of expression (Figure S1) and do not suffer from the effects of crosstalk if used together [19], [20] (Figure S2). For strain W3, the *yddG* gene was cloned into vector pET17 behind the arabinose-inducible promoter *P_BAD_*, which is henceforth referred to as pAK1 (Figure 1B). For strain Y3, *trpEDfbr* was inserted into the pPro24 vector behind the propionate-inducible promoter *P_prpB_*, which resulted in vector pAK5 (Figure 1B). Y3 has also been labeled with yellow fluorescent protein (YFP) to allow tracking of the co-culture composition (Methods).

### Effects of inducers on metabolite secretion and growth properties of engineered strains

To verify the effect of increased metabolite secretion induced by the genetic circuit constructed above, we performed a bioassay [6] to determine the amount of Trp and Tyr in W3 and Y3 monocultures. Each of these engineered strains was grown alone in the minimal medium with and without the corresponding inducer. The culture supernatants were then harvested at times corresponding to the early, middle, and late stages of the exponential growth phase. A test strain, either a Tyr or Trp auxotroph, was then grown on the sterile-filtered supernatant to determine the concentration of the secreted amino acid using a similarly derived calibration curve (Methods). As summarized in Table 1 and Table S2, we observed that, overall, addition of the inducer did increase the secretion of Trp or Tyr when each strain was grown in mono-culture. More specifically, for strain Y3, when the propionate inducer concentration was raised from 0 to 40 mM, the amount of secreted Trp per cell at the end of exponential growth decreased slightly at first and then more than tripled, leading to a maximum Trp concentration of 142±9 μg/L in the supernatant. Interestingly, the amount of Tyr secreted by strain W3 was over 100 fold higher, in the range of a few to several tens of mg/L in the supernatant. When the arabinose inducer was added, the amount of extracellular Tyr accumulated in the mono-culture became so high that it was detectable starting in the early exponential growth phase and steadily increased both as growth proceeded and as the inducer concentration was raised to 0.15%.

**Table 1:** Culture-averaged secretion of Tyr and Trp in mono-culture growth experiments.

Secreted Metabolite	Culture	Time point		
		Early	Middle	End
Trp/OD [μg/L*OD]	Y3	N/D	N/D	95.1±33.2
	Y3+10 mM NaProp	N/D	N/D	63.2±2.8
	Y3+40 mM NaProp	N/D	N/D	**326±21**
Tyr/OD [mg/L*OD]	W3	N/D	N/D	9.77±0.08
	W3+0.08% ara	9.67±0.30	40.0±0.8	56.2±5.8
	W3+0.15% ara	57.5±0.5	**>200**	**>77**

Over the course of growth, W3 secretes more Tyr per cell with the addition of arabinose and Y3 secretes more Trp per cell with the addition of NaProp. Supernatants were harvested at the early, middle stages, and the end of the exponential growth phase. Note the different units of Trp and Tyr (i.e. μg/L*OD vs. mg/L*OD), which reflect the vast difference between the metabolite secretion capacities of the two strains.

In addition to the effect on metabolite secretion, it is also possible that inducing the genetic circuit constructed above may change the growth property of the engineered strains, in particular their nutrient uptake capabilities. For instance, since YddG can also export Trp to some degree [11], we suspected that the expression of this protein on the pAK1 vector may decrease its affinity for Trp and thereby increase the K_m_ value of W3 for the molecule. Therefore, for each strain, we conducted a set of flask experiments to evaluate the inducer's effect on the strain's maximum growth rate and affinity for its essential metabolite (see Methods for details). It was found that for each strain, the corresponding inducer had a negative effect on the maximum growth rate (Figure S3). In particular, the observed growth rate of W3 and Y3 decreased from about 0.6 1/hr to 0.2–0.3 1/hr when the inducer of arabinose or propionate was added. Additional experiments at saturated Trp or Tyr concentrations confirmed these observations, including the unusual trend that the maximum growth rate of strain W3 decreases and then slightly recovers when the arabinose concentration increases (Figure S3A). In addition, by comparing the growth of various strains at saturated Trp or Tyr concentrations, we found that the growth rate decrease of W3 is caused by the pAK1 plasmid, whereas the growth of Y3 is significantly reduced due to the over-expression of the *trpEDfbr* genes on plasmid pAK5 as well as slight inhibition by the propionate inducer (data not shown). This substantial growth decrease of the individual strains caused by induction of the cross-feeding circuit, termed metabolic burden, would have a significant impact on the co-culture's property, as we will discuss later in this paper. On the other hand, with the addition of inducers, the affinity parameters of the engineered strains appeared to have remained at comparable values, in the ranges of 2–4 µg/L Trp for W3 and 5–8 µg/L Tyr for Y3.

### Consortium growth and composition

**Figure 2:**
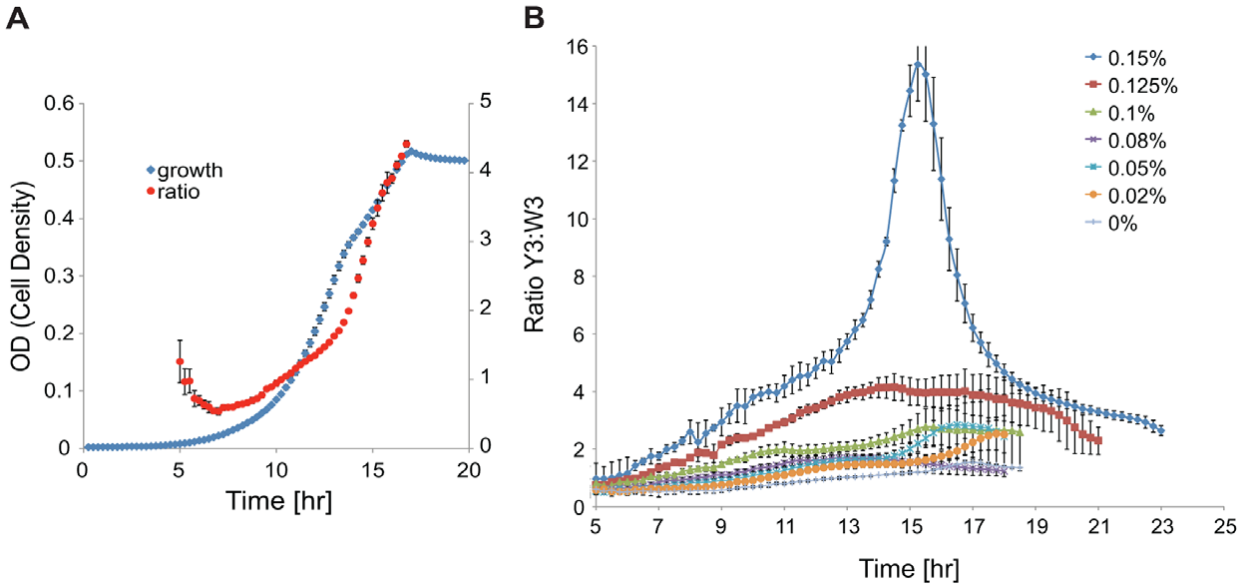
Co-culture growth and ratio dynamics: baseline and with tuning. (**A**) Co-culture density (measured by OD_600_) and Y3∶W3 ratio during growth in minimal medium without inducers. The ratio measurement is shown only for the exponential growth phase because the YFP calibration is not reliable after the cells enter the stationary phase. (**B**) An example of Y3∶W3 ratio dynamics at various arabinose concentrations. Propionate concentration was held at 20 mM. Only the exponential growth phase is shown. Each curve represents the mean of 4 replicates. Note: the secondary y-axis label in (**A**), Ratio Y3∶W3, is also the label for the primary y-axis in (**B**).

The Trp and Tyr auxotroph pair constructed above was first grown in M9 minimal medium for examination of the baseline property without arabinose or propionate (Figure 2A). The growth rate of the co-culture, inoculated with a 1∶1 strain ratio, was found to be 0.45±0.004 1/hr. To determine the ratio (Y3∶W3) of the two auxotrophs, a constitutively expressed YFP gene was integrated into the chromosome of strain Y3. By combining total OD and YFP measurements, we were able to obtain the ratio of the two strains during growth (Methods), however it was observed that the co-culture was not reaching a steady composition (Figure 2A) as predicted by the model described above. This might be because the co-culture had entered the stationary phase before reaching the pseudo steady state. Further experiments revealed that the co-culture exhibits various ratio dynamics when the inducers are added. An example is shown in Figure 2B, in which arabinose was added at different concentrations when propionate was held constant. Depending on the concentration of arabinose, the ratio could monotonically change to a final steady value or exhibit more nonlinear behavior. After observing these complex dynamics, we decided that instead of choosing one value to represent the co-culture “ratio” it would be better to examine the Y3∶W3 ratio in the middle and at the end of the exponential growth phase, henceforth referred to as the mid-exponential and end-exponential ratios, respectively. At the baseline without inducers, the mid-exponential Y3∶W3 ratio was found to be 0.66±0.02, and the end-exponential ratio to be about 4.41±0.05.

**Figure 3:**
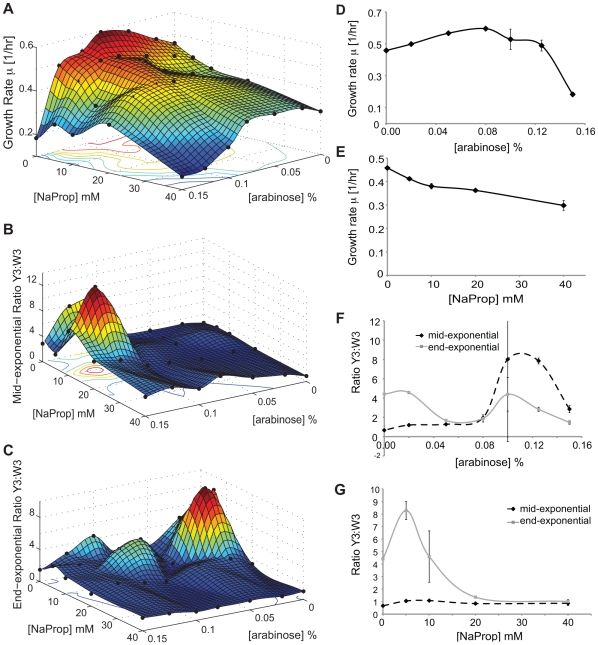
Range of co-culture growth rate and composition regulated by two inducers. Co-culture experiments were conducted over a grid of inducer concentrations. The experimental results (black dots) were then interpolated using Matlab to create a three-dimensional surface. Range of co-culture growth rate (**A**), mid-exponential Y3∶W3 ratio (**B**), and end-exponential Y3∶W3 ratio (**C**) that can be achieved using this circuit. (**D, E, F, G**) Edges of the 3D surfaces. Effect of tuning arabinose and propionate on the growth rate (**D, E**) and Y3∶W3 ratios (**F, G**). (**D, F**) 0 mM propionate (**E, G**) 0% arabinose. Both the mid-exponential and end-exponential ratio were determined using the YFP calibration. Experimental data are given in Data S1.

By simultaneously adding the two inducers that regulate the metabolic cross-feeding circuit, we were indeed able to change the growth rate and composition of the synthetic consortium (Figure 3). Overall we achieved a fairly large range of co-culture growth rate (0.16–0.59 1/hr, Figure 3A), mid-exponential ratio (13 to 0.6, Figure 3B), and end-exponential ratio (9.7 to 0.9, Figure 3C). Adding the inducers, however, does not affect these co-culture properties in the simple manner our basic model suggested. Instead, the relationship between the co-culture growth rate and inducer concentrations is highly nonlinear, which can be illustrated with the two edges where only one inducer is involved. When only arabinose is added (Figure 3D), increasing arabinose caused the co-culture growth rate to increase from 0.45 1/hr to 0.58 1/hr at 0.08% arabinose, supposedly due to increased secretion of Tyr by strain W3. Further increasing the arabinose concentration to 0.15%, unexpectedly, decreased the co-culture growth rate sharply to 0.20 1/hr. On the other hand, when only propionate is added, the co-culture growth rate monotonically decreased from 0.46 to 0.30 1/hr (Figure 3E).

We also observed highly nonlinear dependence of the co-culture composition on inducer concentrations (Figure 3B&C). Interestingly, when two edges of the 3-D plots were examined more closely, tuning the W3 strain through arabinose seemed to have more of an effect on the mid-exponential ratio (Figure 3F), whereas tuning the Y3 strain through propionate seemed to have more of an effect on the end-exponential ratio (Figure 3G).

The above relationships between consortium properties (i.e. growth rate and strain ratio) and inducer concentrations we have observed are quite complex and nonlinear. This is likely due to multiple factors affecting the growth dynamics of our system, most importantly each inducer's double effect of increasing metabolite secretion while decreasing growth.

**Figure 4:**
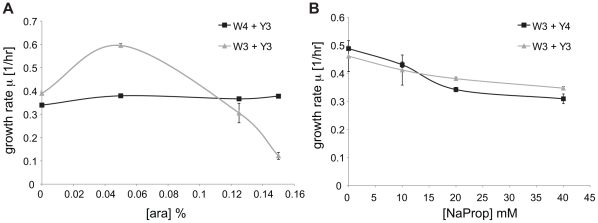
Negative control experiments. (**A**) Addition of arabinose has no effect on the growth rate of the negative tryptophan control strain (W4) and the positive tyrosine strain (Y3). (**B**) The growth rate of both cultures is decreased with the addition of increasing amounts of propionate, but the growth rate decreases less with the addition of the cross-feeding genes. For both (**A**) and (**B**) the negative control strain is either W4 or Y4. See Table S1 for the complete strain genotypes.

To verify the effect of regulated cross-feeding on the co-culture growth, we carried out two sets of negative control experiments in which the cross-feeding auxotroph pair was exactly the same as W3 - Y3 except that either the *yddG* gene in W3 or the *trpEDfbr* genes in Y3 was replaced by a negative control gene that does not participate in the cross-feeding circuit (i.e. CFP or GFP). It was observed that when *yddG* was not over-expressed in the tryptophan auxotroph, adding arabinose did not affect the co-culture growth (Figure 4A). Furthermore, at low concentrations of arabinose, the negative control pair grew slower than the pair with the full circuit. On the other side, when *trpEDfbr* was not expressed in the tyrosine auxotroph, adding propionate reduced the co-culture growth rate substantially (Figure 4B). Notably, the pair with the full circuit grew faster than this negative control pair when propionate is at medium to high concentrations. These results indicate that tuning the target genes in the designed circuit is indeed regulating the cross-feeding and hence the co-culture growth. Clearly there is a side effect of metabolic burden from this circuit, and regulation of the co-culture is more effective when the enhanced cross-feeding is not over-shadowed by the burden (for example when the arabinose concentration is not too high and propionate is at medium to high levels).

To understand further how the two strains W3 and Y3 interact in this co-culture, we also attempted to measure the amounts of Trp and Tyr in the co-culture supernatants at the grid “corners”, namely the arabinose and propionate concentration combinations of (0%, 0 mM), (0.15%, 0 mM), (0%, 40 mM), and (0.15%, 40 mM). Using the same bioassay employed for the monocultures (Methods), we found that throughout the course of co-culture growth, the concentrations of Trp and Tyr remained below detectable levels (Trp: ∼10 μg/L; Tyr: ∼0.1 mg/L). This was in sharp contrast to their obvious accumulation in the monocultures and was in line with what we had initially hypothesized, i.e. that each amino acid is the limiting nutrient for the corresponding auxotrophic strain. Given the high affinity of each auxotroph for its required amino acid, with K_m_ in the range of several µg/L (Figure S3), it is very likely that the amino acid is taken up by the auxotrophic strain as soon as the molecule is secreted by the partner strain and hence does not accumulate in the supernatant. In fact, based on the observation that in a co-culture each strain's growth rate was largely below its maximum value associated with the saturated amino acid concentration (∼10 μg/L Trp for W3 and ∼50 μg/L Tyr for Y3, Figure S3), we could infer that the Trp/Tyr concentrations were indeed very low, up to a few μg/L for Trp and several tens of μg/L for Tyr.

### Programming the synthetic consortium with the Design Space

**Figure 5:**
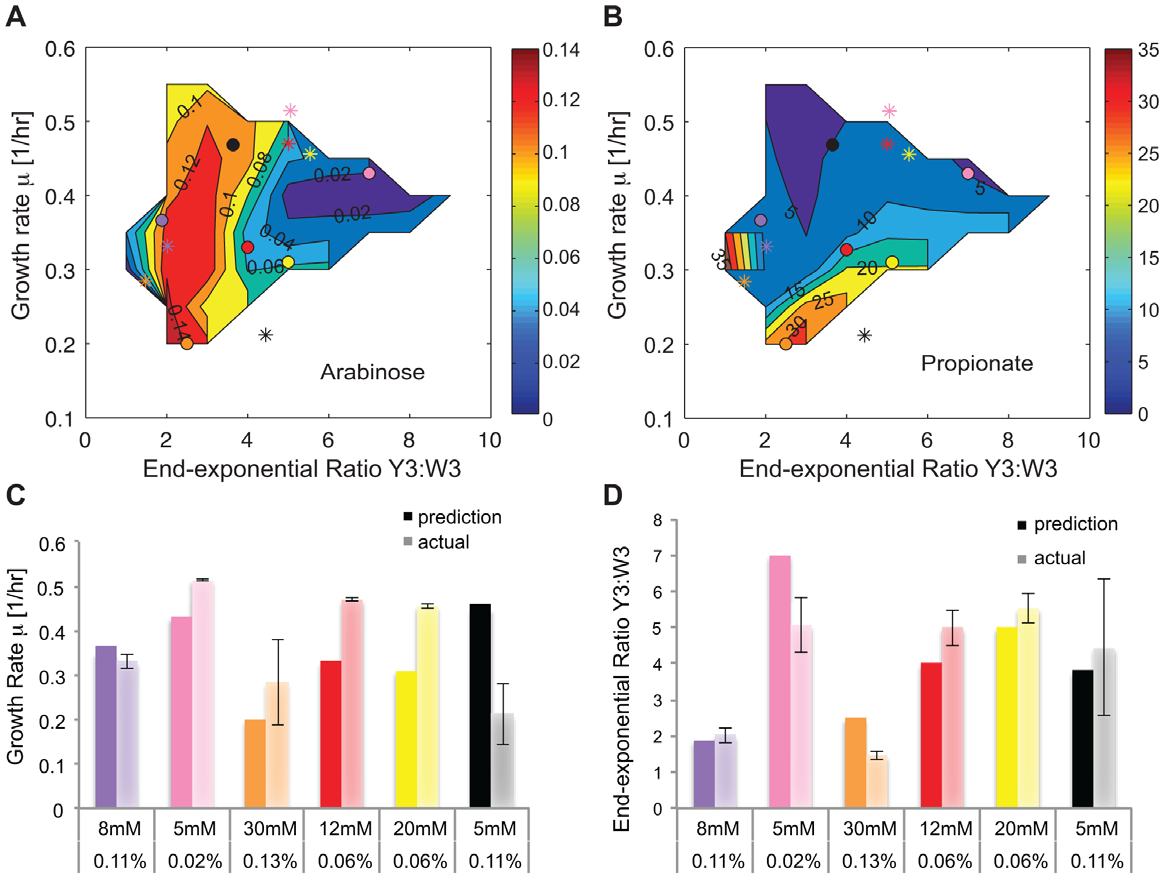
Design space and testing. (**A, B**) By inverting the relationships of growth rate/strain ratio vs. inducer concentrations, a design space was generated to represent the two-dimensional space of achievable growth rates and strain ratios, and to determine the arabinose (**A**) and propionate (**B**) concentrations for a desired growth rate and end-exponential ratio combination. The colored circles are “prediction” points and the asterisks of the same color are the “actual” results of using that combination of arabinose and propionate in the co-culture. The colors denote inducer combinations: purple (0.11%, 8 mM); pink (0.02%, 5 mM); orange (0.13%, 30 mM); red (0.06%, 12 mM); yellow (0.06%, 20 mM); black (0.11%, 5 mM). (**C, D**) Comparing the predicted and actual outcome for growth rate (**C**) and end-exponential ratio (**D**) in bar graph form; the predictions are in darker colors and the actual (experimental) results are in lighter ones. Error bars: ± standard deviation. The mid-exponential ratio design space is shown in Figure S4. Experimental data are given Data S1.

To utilize the above results for programming the consortium, we reversed the relationships of growth rate/strain ratio versus inducer concentrations and defined a design space represented by two contour plots (see Methods for details). For achieving a specific growth rate and end-exponential ratio combination, Figure 5A and Figure 5B show what arabinose and propionate concentrations shall be used, respectively. Similarly, Figure S6A&B can be used to determine the inducer concentrations for achieving a specific growth rate and mid-exponential ratio combination. Due to the high nonlinearity of the dependence of growth rate and strain ratio on inducer concentrations (Figure 3), the design spaces are also very nonlinear and exhibit irregular shapes (Figure 5A&B, Figure S6A&B). For both arabinose and propionate, the two-dimensional contour plot has both “steep” areas featuring tight contour lines, where substantially varying the inducer concentration is required to change the growth/ratio, and almost “flat” regions, where small changes of inducer concentration correspond to large changes of growth/ratio. Additionally, higher concentrations of arabinose appear to produce lower end-exponential ratios and variable growth rate, while higher propionate concentrations seem to produce lower growth (except the small region on the left, Figure 5B).

To test the accuracy of the design space, a “prediction” point was first selected at the same position in both plots of Figure 5A&B, which would correspond to a particular growth rate and ratio. For example, the yellow circles correspond to a growth rate of ∼0.31 1/hr and an end-exponential ratio of ∼5. At this specific point, using the contour lines, the inducer concentrations can be estimated to be 0.06% arabinose (Figure 5A) and 20 mM propionate (Figure 5B). The auxotroph pair was then grown under this specific combination of inducers and the resulting growth rate and ratio (the “actual” data) were compared with the “predicted” values. Results from six such tests are illustrated in Figure 5 (panels A&B: circles – predictions, asterisks – actual values from experiments; panels C&D: bar graph comparisons). For the growth rate, two out of these six tests showed good agreement between predicted and actual outcome (within two standard deviations, Figure 5C: 8 mM, 0.11%; 30 mM, 0.13%); for the end-exponential ratio, four of them are reasonably accurate (within two standard deviations, Figure 5D: 8 mM, 0.11%; 12 mM, 0.06%; 20 mM, 0.06%; 5 mM, 0.11%). These results confirm our expectation that the steeper regions of the design space are more accurate, while the flatter regions are more difficult to target. It is also worth noting that the contours could explain, at least partially, the deviation of the actual outcome from the prediction. For example, for the inducer concentration of 0.06% arabinose and 12 mM propionate, the predicted and actual values of growth rate are significantly different (Figure 5A, red circle and asterisk), but the plot illustrates how the point have moved around the arabinose surface along the 0.06% contour.

## Discussion

As described above, we have constructed a proof-of-principle biological circuit to regulate the growth rate and composition of a two-member *E. coli* consortium based on tunable symbiosis. The resulting co-culture is able to achieve a continuous range of growth rate and composition; in addition, we show that the system can be “programmed” reasonably well for desired growth rate or strain ratio. The symbiotic scheme (two auxotrophic strains cross-feeding amino acids) has been proposed and examined in previous work, most notably with the yeast system by Shou and coworkers [6]. Building on this basic concept in our work here, we have devised a novel approach for continuously *tuning* two important properties of synthetic consortia: the growth rate and community composition. Whereas previous work largely focused on using synthetic circuits to investigate the mechanism of microbial interactions such as mutualism, the main objective of this study has been developing a tool for engineering a synthetic microbial community, which can be deployed in various applications.

The main issue of our current system is that the metabolic burden partially masks the cross-feeding benefits with regard to the growth rate. This obstacle could potentially be overcome by using plasmids of lower copy number, by modifying the promoters to achieve more appropriate expression levels, or by transferring the system to the chromosome. On the other hand, since the gene expression level would be lower, the effect of activating the circuit might also become smaller. Nevertheless, we expect that eliminating or reducing the metabolic burden would lead to larger and more predictable ranges of growth rate and strain ratio upon addition of inducers. This would also improve the quality of the design space, which ideally would exhibit monotonic relationships between inducer levels and desired growth/ratio (as would be expected for the design space corresponding to our basic model) and hence provide better estimates of inducer concentrations for generating the desired co-culture property.

We also want to point out that our measurement of consortium composition (i.e. strain ratio), via a combination of absorbance and fluorescence readout, is not ideal and occasionally showed large variation among replicates when the ratio deviates substantially from one and changes rapidly over time. In addition to inherent variability of the biological system (e.g. due to gene expression), another possible source could be the population variances in YFP expression and maturation. In our current system, GFP variant eYFP is constitutively expressed on the chromosome in the tyrosine auxotroph but its expression is not very high. Furthermore, YFP in its native state has a slow maturation time [21], which may be causing the inaccuracy and large variation. Using another fluorescent protein with higher signal/noise ratio and faster maturation time could potentially reduce or eliminate these issues and hence improve the measurement. It will also be worth exploring in the future alternative methods, such as qPCR and fluorescence-activated cell sorting (FACS), to achieve better accuracy.

Finally, we would like to emphasize that the approach reported in this work for regulating and programming a two-member synthetic microbial consortium and its extensions could be readily transferred to more complex systems consisting of different microbial strains or species. Two key components are required to construct such a regulatory circuit. First, the two consortium members need to form an inter-dependent relationship. Part of this inter-dependence might already exist when a synthetic microbial consortium is assembled [22], [23]. If a complete cross-feeding loop is not in place, genetic manipulation such as the gene deletions we conducted in this work to generate auxotrophs will be needed. Second, genes that can affect the export of cross-fed metabolites need to be regulated, which can be achieved by various means, for instance through the usage of chemically inducible promoters as illustrated in this work. The resulting tunable microbial consortia can potentially be utilized for many applications. For example, complete and efficient co-fermentation of hexose and pentose sugars is one of the major obstacles in effectively converting lignocellulosic biomass into fuels [24]. Existing strategies to optimize the sugar utilization using a bacterial co-culture include delaying the inoculation time of one of the strains or changing the inoculation ratio [25]. Tuning the composition of the co-culture during growth might be easier and more efficient than either of the previous strategies.

## Materials and Methods

### Basic co-culture model

An ODE system was formulated to describe the growth dynamics of a pair of auxotrophs that cross feed each other, as shown in Figure 1A (Reppas, Lin, *et al.*, manuscript in preparation). Using Monod kinetics for cell growth on a limiting nutrient, the governing equations are as follows:
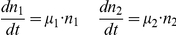


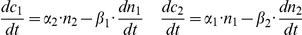


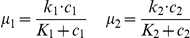
where n_1_ and n_2_ are the cell densities of the two auxotrophs (gDM/L), c_1_ and c_2_ are the concentrations of the cross-fed metabolites (mM), α_1_ and α_2_ are the auxotrophs' export rate of the metabolites (µmol/gDM-hr), β_1_ and β_2_ are their cellular requirement for the essential metabolites (µmol/gDM), respectively. To model a batch co-culture in the minimal medium without supplementation of the cross-fed metabolites, c_1_ and c_2_ are set to zeros as the initial condition. Computer simulation showed that given sufficient time, the co-culture would reach a “pseudo steady state” with two characteristics: i) the ratio of the two auxotrophs remains constant, which indicates that they grow at the same rate; and ii) concentrations of the two cross-fed metabolites remain constant. By making use of these conditions, we can readily derive the following analytical formula to describe the system's growth rate and composition at this pseudo steady state, as functions of the auxotrophs' properties.
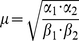
(1)

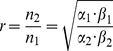
(2)It therefore follows that at this state, the growth rate of the co-culture, which is the same as that of each auxotroph, and the ratio of the two auxotrophs are determined solely by the α and β parameters.

### Auxotroph construction and YFP addition

A Trp auxotroph, strain W1, was constructed via P1 transduction of the *trpE* gene replaced with a KanR cassette from strain JW1469-1 (Keio collection, http://ecoli.naist.jp/gb6/Resources/deletion/deletion.html) into wild-type *E. coli* K12 MG1655 [26]. P1-facilitated gene deletion was repeated in strain W1 with the *tyrR* gene (JW1316), to obtain strain W2, a double knockout, and was also used to knock out the *tyrA* gene (JW2581) to create the Tyr auxotroph, strain Y1. The *yfp* gene was introduced into strain Y1 via the same method (host strain DS1-Y was obtained from the Balaban group) and was integrated into the *intC* locus with a *cat* selection cassette (strain Y2). This gene is under control of two λP_R_ promoters [27]. The *trpE* and *tyrA* gene knockouts were confirmed via colony PCR, as well as phenotypically by growing the strains alone in unsupplemented M9 medium after each genetic manipulation. None of the auxotrophs grew without its partner strain (data not shown).

### Plasmid construction

The arabinose-inducible promoter P_BAD_ was amplified from strain BW31003 (CGSC #8183) digested with restriction endonucleases NdeI and HindIII, and ligated to the pET17 expression vector (Novagen). The *yddG* gene was amplified from wild-type *E. coli* MG1655, digested with XhoI and HindIII, and ligated to the pET17-P_BAD_ construct giving vector pAK1. This vector was transformed into the W2 strain, producing strain W3. The *trpEDfbr* gene cassette was synthesized (Geneart) and then cut with SalI and EcoRI and ligated to the pPro24-gfp vector behind the propionate-inducible promoter P_prpB_ with GFP removed (Addgene). This vector was then transformed into strain Y2, giving strain Y3. Both plasmids confer ampicillin resistance.

### Co-culture composition determination using YFP

Minimal M9 medium containing 0.2% glucose was used in all experiments. For circuit induction, either arabinose (20% w/v stock) or sodium propionate (1 M stock) was used at the indicated concentration. Frozen stocks were inoculated into rich LB medium and grown overnight to saturation. The cells were then washed and diluted by 1∶800 into fresh minimal M9 medium and the appropriate inducers were added. Cultures were then pipetted onto a 96-well microplate (Grenier) to a volume of 200 µl per well. Unless otherwise stated, four replicates were conducted per sample. A Biotek Synergy 2 microplate reader was used to monitor co-culture growth and composition over time via reading the absorbance at 600 nm and the YFP fluorescence using filters for excitation (485/20) and emission (528/20). For each microplate growth experiment, calibration between the Y3 strain OD_600_ and the fluorescence (FL) was obtained by plotting the FL vs. the OD_600_ and fitting the data with a linear model (see Figure S5 for a sample calibration curve). The slope was then used to determine the Y3 density in the co-culture (note that we could have also considered the “fluorescence” of the W3 strain, but it did not significantly change the results). The W3 density was obtained by subtracting the Y3 density from the total OD_600_. The ratio Y3∶W3 is then equal to the density of Y3 divided by the density of W3. Cultures were grown for 48 hours at 37°C with shaking and measurements were taken every 15 minutes.

To validate our method for determining the co-culture composition using YFP, we tested four co-culture samples and compared ratio results obtained via the above method and differential plate counting (i.e. using viable cell counts from minimal medium petri dishes supplemented with Trp or Tyr). The co-cultures were grown and monitored on a microplate reader. Based on manual inspection of the growth curve, each co-culture was stopped either in the middle or at the end of exponential growth whereupon samples were extracted for differential plate counting (note that it is virtually impossible to continue a microplate growth experiment once it is stopped, due to technical complications). Out of the four tested samples, three showed reasonable agreement between the ratio results from the two different methods (Figure S6). In addition, we observed that when the ratio is close to one and does not change rapidly, the result from YFP calibration is in excellent agreement with that from plate counting and the error bar is very small. However, when the ratio deviates substantially from one and fluctuates over time (e.g. Figure 2B, the condition of 0.15% arabinose), the result from YFP calibration tends to be much less accurate and the error bar becomes much larger.

### Measurement of Trp and Tyr concentrations in mono- and co-culture supernatants

Concentrations of Trp and Tyr in mono- and co-cultures were estimated using a bioassay similar to one previously reported [6]. The cultures were grown in 10 ml M9 media in 50-ml falcon tubes. The monocultures were also supplemented with saturating amounts of Trp and Tyr (40 µg/ml). Inducers were added as needed. 1-ml samples were harvested at various time points over the course of growth corresponding to the early, middle and late exponential growth phases. The OD_600_ of the cultures was monitored over time to identify these points. The 1-ml samples were centrifuged at 12,000 rpm and the supernatants were sterile-filtered and stored at −20°C. An auxotrophic test strain, W1 or Y1, was grown on the sterilized culture supernatants supplemented with concentrated M9 (400 µl supernatant and 100 µl 5X M9). The maximum OD_600_ reading was then used to determine the initial Trp or Tyr concentration in each supernatant, according to a calibration curve. The Trp and Tyr calibration curves were prepared as follows. Standard dilutions of Trp or Tyr stocks were made with M9 media and then the corresponding auxotrophic test strain was grown on each standard for about 48 hours on a microplate reader at 37°C with shaking. OD_600_ measurements were taken every 15 minutes, and the maximum OD_600_ of each sample was used to generate the calibration curve.

### Measurement of Trp and Tyr Affinities

Each strain (either W3 or Y3) was grown alone in M9 with specified amounts of Trp or Tyr at various inducer concentrations to determine the effect of the inducer on the strain's affinity and maximum growth rate. More specifically, cells were first grown overnight in minimal M9 media with saturating amounts of Trp or Tyr (40 µg/ml). The cells were then washed three times in M9 and diluted to a final density of ∼1000 cells/ml in 50 ml M9 in a 250 ml flask supplemented with the desired metabolite and inducer concentrations. The cultures were grown at 37°C in a shaking water bath and the initial growth rate was measured via plate counting. The range and spacing of sampling times were dependent on the expected growth rate of the culture. For each initial Trp or Tyr concentration, after obtaining the cell counts, the exponential growth rate and goodness of fit (the error bar shown at each point in Figure S3) were determined using Excel. Finally, the Matlab curve-fitting tool was used to fit each curve of growth-rate vs. Trp/Tyr concentration to a Monod function and to obtain the μ^max^ and K_m_ values.

### 3D surface and design space plots

Experimental 3D surface results and design spaces were plotted using MATLAB. For the growth and ratio results, interpolation was carried out (griddata) and the results were used to plot the 3D surfaces. To create the design spaces, griddata was again used to obtain points across a continuous space and then a closed contour plot (contourf) was created using the interpolated results.

## Supporting Information

Figure S1 **Relationship between gene expression level, measured by GFP/OD_600_, and inducer concentration for the P_BAD_ (A) and P_prpB_ (B) promoters.** Inducers were added to cultures of single strains expressing GFP behind either the arabinose- or propionate-inducible promoters. (**A**) Strain W5 and (**B**) strain Y4. Growth and fluorescence data were taken from the end of exponential growth phase when the expression level was constant.
(EPS)

Figure S2 **P_BAD_ (A) and P_prpB_ (B) do not suffer from cross talk between the promoter and the other's inducer so they can be used together in co-culture.** P_prpB_ seems to be leakier than P_BAD_. (**C**) Close-up of (**A**), no change with varying arabinose. See Table S1 for complete strain genotype. The strains used were all tyrosine auxotrophs since only the effect of each inducer on GFP expression from the opposing promoter was being investigated (in this particular experiment).
(TIFF)

Figure S3 **Growth rates of W3 and Y3 at various Trp and Tyr concentrations.** The maximum growth rates and affinity of W3 for Trp (**A**) and of Y3 for Tyr (**B**) were measured under inducing and non-inducing conditions. The Matlab curve-fitting tool was used to fit each growth curve to a Monod function and to obtain the μ^max^ and K_m_ values. The error bar at each point on the growth curve represent the goodness of the exponential growth curve fit. The R^2^ values for each Monod fit are as follows: W3 - 0%, 0.92; 0.08%, 0.82; 0.015%, 0.87; and Y3 - 0 mM, 0.96; 20 mM, 0.88; 40 mM, 0.78.
(TIFF)

Figure S4 (**A, B**) **Mid-exponential ratio design space 2D plots.** Using Matlab, two-dimensional design spaces were generated for arabinose (**A**) and for propionate (**B**) using the growth rate and mid-exponential ratio data. The colored circles are “prediction” points, and the asterisks of the same color are the actual results of using that combination of arabinose and propionate. The colors denote the same inducer combination between (**A**) and (**B**): white (0.13%, 0 mM); purple (0.08%,12); light blue (0.11%, 25); black (0.10%, 5 mM); yellow (0.11%, 20 mM); pink (0.12%, 8 mM). (**C, D**) Mid-exponential ratio design space predictions and results. Six different arabinose and propionate combinations were tested. The predictions are the darker shade and the actual (experimental) results are the lighter shade. (**C**) Growth rate predictions and outcome. (**D**) Ratio predictions and outcome. Each prediction and result in (**C**) has a corresponding representation in (**D**).
(TIF)

Figure S5 **Sample YFP calibration.** Four replicates of each of K12 and Y3 were averaged, and then the YFP FL readout was plotted against the OD_600_. The calibrations are linear during the exponential phase of growth, which is shown in the graph. Excel was used to fit the data using linear regression.
(TIF)

Figure S6 **Comparison of Y3∶W3 ratio results determined using YFP calibration vs. plate counting.** For each co-culture condition, 4 wells (replicates) were used to determine the Y3∶W3 ratio using the YFP calibration method. After a certain period of time, the microplate reader was stopped and the 4 wells were pooled and plated on 3 or 4 M9 minimal plates with Trp and 3 or 4 M9 minimal plates with Tyr. Each co-culture was diluted appropriately to give 30–300 colonies per plate for accurate counting. The ratio was then calculated using all combinations (9–16) of the cell count of Y3 from Tyr+ plates and that of W3 from Trp+ plates. Note that these conditions did not match exactly those for the mid and end-exponential ratios in Figure 3, since the microplate reader was stopped at time points that allowed us to sample several cultures simultaneously and only corresponded to approximately the middle and end of exponential growth.
(TIF)

Table S1 **Complete strain list.**
(DOC)

Table S2 **Trp concentrations in the supernatant of Y3 growing alone with either 0, 10, or 40 mM NaProp and Tyr concentrations in the supernatant of W3 growing alone with 0, 0.08, or 0.15% arabinose over time.**
(DOCX)

Data S1 **Experimental data used to create** **Figure 3** **and** **Figure 5** **.**
(XLS)
