# Supplementary figures and images for: A Programmable Escherichia coli Consortium via Tunable Symbiosis
Source: PLoS One. 2012 Mar 30;7(3):e34032. doi: 10.1371/journal.pone.0034032 (PMC3316586; doi:10.1371/journal.pone.0034032)

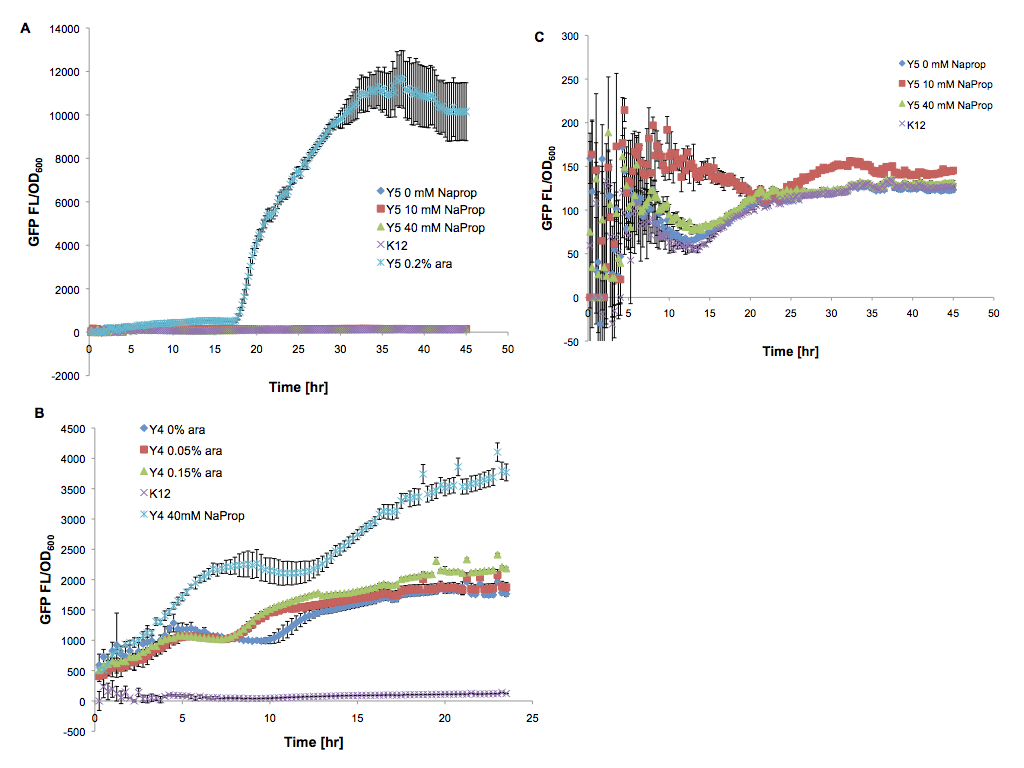

Supplement: Figure S2 — PBAD (A) and PprpB (B) do not suffer from cross talk between the promoter and the other's inducer so they can be used together in co-culture. PprpB seems to be leakier than PBAD. (C) Close-up of (A), no change with varying arabinose. See Table S1 for complete strain genotype. The strains used were all tyrosine auxotrophs since only the effect of each inducer on GFP expression from the opposing promoter was being investigated (in this particular experiment). (TIFF) [file pone.0034032.s002.tiff]

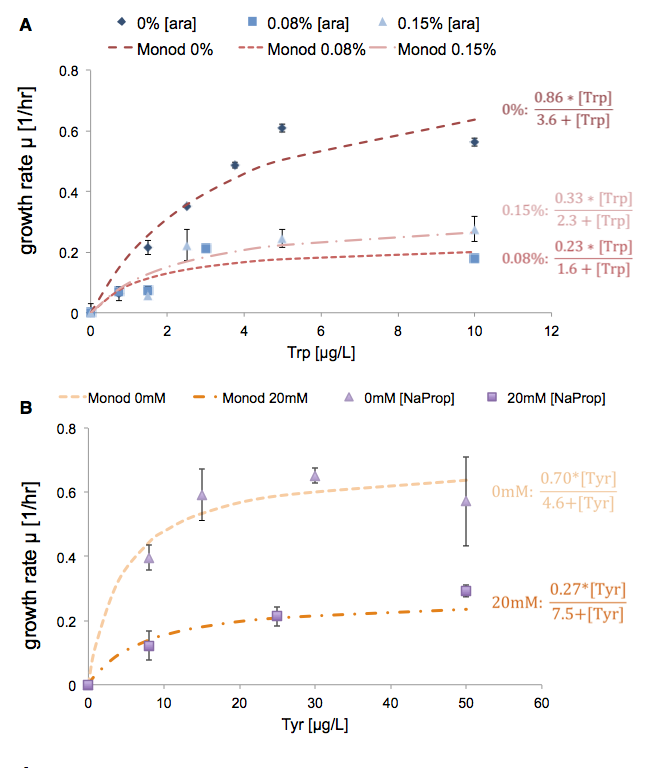

Supplement: Figure S3 — Growth rates of W3 and Y3 at various Trp and Tyr concentrations. The maximum growth rates and affinity of W3 for Trp (A) and of Y3 for Tyr (B) were measured under inducing and non-inducing conditions. The Matlab curve-fitting tool was used to fit each growth curve to a Monod function and to obtain the μmax and Km values. The error bar at each point on the growth curve represent the goodness of the exponential growth curve fit. The R2 values for each Monod fit are as follows: W3 - 0%, 0.92; 0.08%, 0.82; 0.015%, 0.87; and Y3 - 0 mM, 0.96; 20 mM, 0.88; 40 mM, 0.78. (TIFF) [file pone.0034032.s003.tiff]

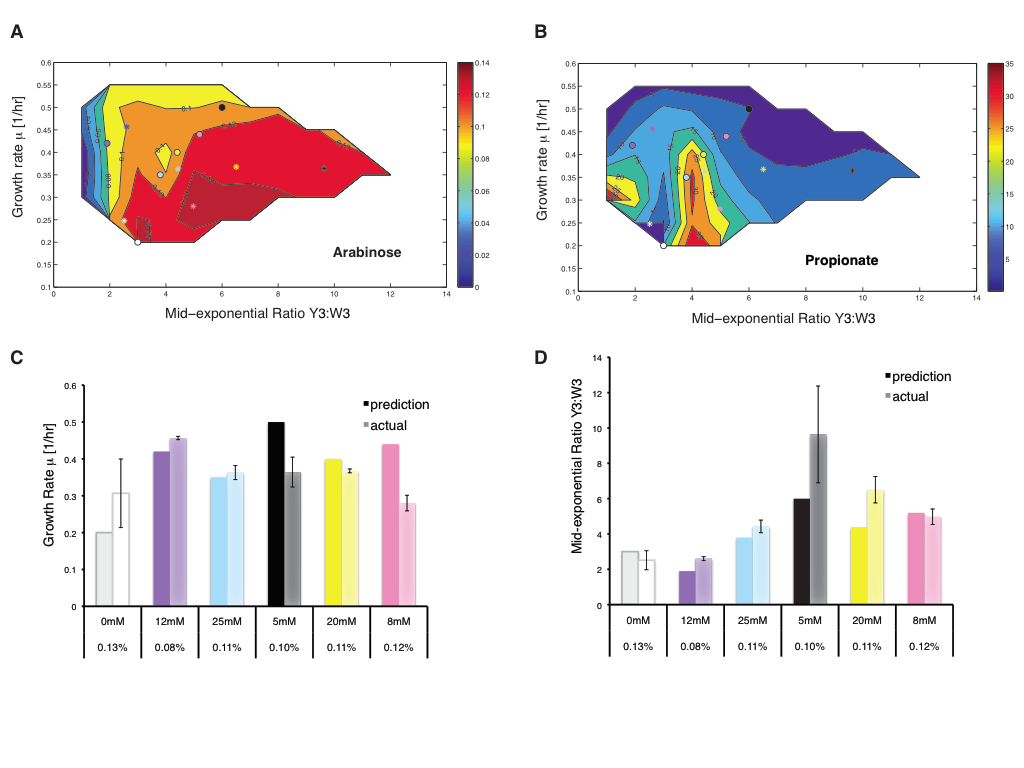

Supplement: Figure S4 — (A, B) Mid-exponential ratio design space 2D plots. Using Matlab, two-dimensional design spaces were generated for arabinose (A) and for propionate (B) using the growth rate and mid-exponential ratio data. The colored circles are “prediction” points, and the asterisks of the same color are the actual results of using that combination of arabinose and propionate. The colors denote the same inducer combination between (A) and (B): white (0.13%, 0 mM); purple (0.08%,12); light blue (0.11%, 25); black (0.10%, 5 mM); yellow (0.11%, 20 mM); pink (0.12%, 8 mM). (C, D) Mid-exponential ratio design space predictions and results. Six different arabinose and propionate combinations were tested. The predictions are the darker shade and the actual (experimental) results are the lighter shade. (C) Growth rate predictions and outcome. (D) Ratio predictions and outcome. Each prediction and result in (C) has a corresponding representation in (D). (TIF) [file pone.0034032.s004.tif]

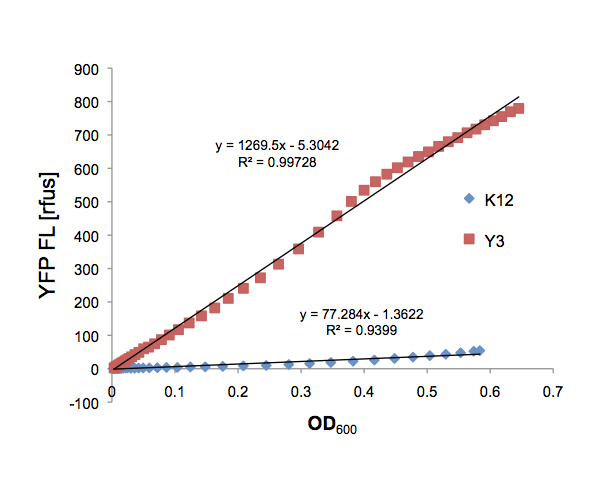

Supplement: Figure S5 — Sample YFP calibration. Four replicates of each of K12 and Y3 were averaged, and then the YFP FL readout was plotted against the OD600. The calibrations are linear during the exponential phase of growth, which is shown in the graph. Excel was used to fit the data using linear regression. (TIF) [file pone.0034032.s005.tif]

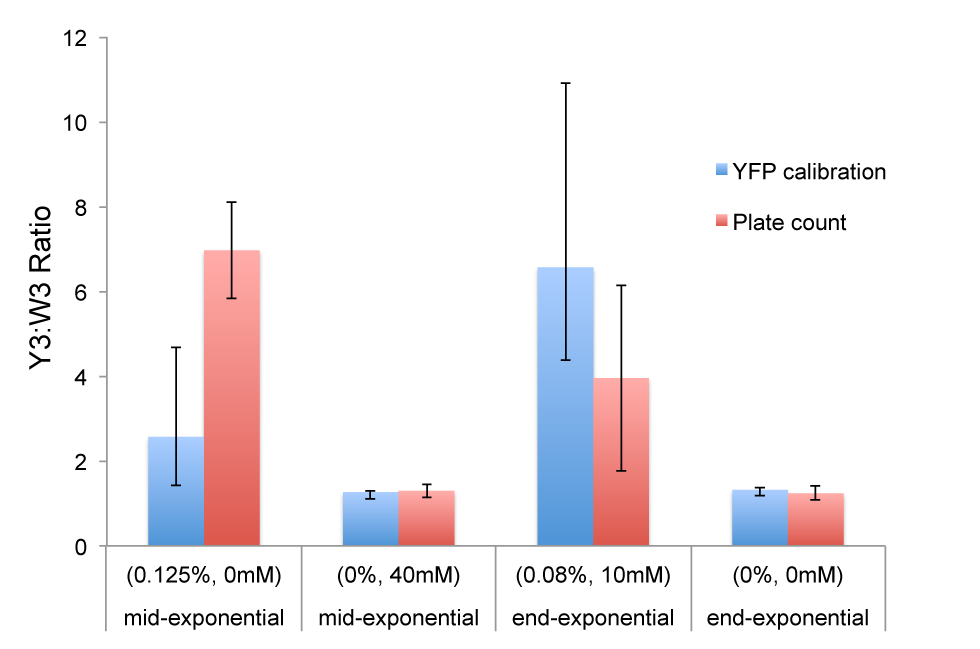

Supplement: Figure S6 — Comparison of Y3∶W3 ratio results determined using YFP calibration vs. plate counting. For each co-culture condition, 4 wells (replicates) were used to determine the Y3∶W3 ratio using the YFP calibration method. After a certain period of time, the microplate reader was stopped and the 4 wells were pooled and plated on 3 or 4 M9 minimal plates with Trp and 3 or 4 M9 minimal plates with Tyr. Each co-culture was diluted appropriately to give 30–300 colonies per plate for accurate counting. The ratio was then calculated using all combinations (9–16) of the cell count of Y3 from Tyr+ plates and that of W3 from Trp+ plates. Note that these conditions did not match exactly those for the mid and end-exponential ratios in Figure 3, since the microplate reader was stopped at time points that allowed us to sample several cultures simultaneously and only corresponded to approximately the middle and end of exponential growth. (TIF) [file pone.0034032.s006.tif]
