# Supplementary material for: A Programmable Escherichia coli Consortium via Tunable Symbiosis
Source: PLoS One. 2012 Mar 30;7(3):e34032. doi: 10.1371/journal.pone.0034032 (PMC3316586; doi:10.1371/journal.pone.0034032)
Supplement: Table S1 — Complete strain list. (DOC) [file pone.0034032.s007.doc]

| **Auxotrophy** | **Name** | **Genotype** | **Vector genes** |
| --- | --- | --- | --- |
| **Tryptophan** | W1 | *K12 ΔtrpE::kanR* | / |
|  | W2 | *K12 ΔtrpE ΔtyrR::kanR* | / |
|  | W3 | *K12 ΔtrpE ΔtyrR::kanR pAK1* | P_BAD_-yddG AmpR |
|  | W4 | *K12 ΔtrpE ΔtyrR::kanR pCFP* | P_BAD_-cfp AmpR |
|  | W5 | *K12 bioA::λRed ΔtrpABCDE::cat pGFP* | P_BAD_-gfp AmpR |
| **Tyrosine** | Y1 | *K12 ΔtyrA::kanR* | / |
|  | Y2 | *K12 ΔtyrA::kanR intC::yfp* | / |
|  | Y3 | *K12 ΔtyrA::kanR intC::yfp pAK5* | P_prpB_-trpEDfbr AmpR |
|  | Y4 | *K12 ΔtyrA::kanR intC::yfp pPro24-gfp* | P_prpB_-gfp AmpR |
|  | Y5 | *K12 bioA::λRed ΔtyrA::cat pGFP* | P_BAD_-gfp AmpR |

**Table S1.**
