# Supplementary material for: A Programmable Escherichia coli Consortium via Tunable Symbiosis
Source: PLoS One. 2012 Mar 30;7(3):e34032. doi: 10.1371/journal.pone.0034032 (PMC3316586; doi:10.1371/journal.pone.0034032)
Supplement: Table S2 — Trp concentrations in the supernatant of Y3 growing alone with either 0, 10, or 40 mM NaProp and Tyr concentrations in the supernatant of W3 growing alone with 0, 0.08, or 0.15% arabinose over time. (DOCX) [file pone.0034032.s008.docx]

**Table S2.**

| **Secreted Metabolite** | **Culture** | **Time point** | | |
| --- | --- | --- | --- | --- |
|  |  | early | middle | end |
| **Trp [μg/L]** | Y3 | N/D* | N/D* | 46.9 ± 16.4 |
|  | Y3 + 10mM NaProp | N/D* | N/D* | 26.4 ± 1.2 |
|  | Y3 + 40mM NaProp | N/D* | N/D* | 142 ± 9 |
| **Tyr [mg/L]** | W3 | N/D* | N/D* | 4.38 ± 0.04 |
|  | W3 + 0.08% ara | 0.174 ± 0.005 | 10.7 ± 0.2 | 21.0 ± 2.2 |
|  | W3 + 0.15% ara | 2.18 ± 0.02 | >31** | >31** |

*: Not detectable, below range of the calibration curve (Trp: ~10 μg/L**;** Tyr: ~0.1 mg/L).

**: Above range of the calibration curve, lower bound indicated.
